# Comparison of Mechanical Properties of Non-ridged Versus Ridged Backslabs in Lower Limb Fractures

**DOI:** 10.7759/cureus.49235

**Published:** 2023-11-22

**Authors:** Muhammad Maaz Gul Kaka Khel, Syed Naveed Mohsin, Faisal Shahzad, Philip Purcell, Amir Siddique, Mahmood Ahmad, Muhammad Shahab

**Affiliations:** 1 Trauma and Orthopaedics, Liverpool University Hospitals Foundation Trust, Liverpool, GBR; 2 Trauma and Orthopaedics, Saint James's Hospital, Dublin, IRL; 3 General Surgery, Cavan General Hospital, Cavan, IRL; 4 Orthopaedics, Mayo Hospital, Lahore, Lahore, PAK; 5 Mechanical Engineering, Centre of Applied Science for Health, Technological University Dublin, Dublin, IRL; 6 Electronic and Mechanical Engineering, Dundalk Institute of Technology, Dundalk, IRL; 7 Trauma and Orthopaedics, Our Lady of Lourdes Hospital, Drogheda, IRL; 8 Trauma and Orthopaedics, Shifa International Hospital Islamabad, Islamabad, PAK; 9 Trauma and Orthopaedics, Royal Bolton Hospital, Bolton, GBR

**Keywords:** non ridged backslab, lower limb cast, plaster cast, load to failure, ridged backslab

## Abstract

Introduction

Lower limb fractures frequently require immobilization with backslabs to promote healing. This study investigates a novel approach involving the incorporation of a single ridge to enhance backslab strength while maintaining cost-effectiveness.

Objective

The aim of this study was to assess the mechanical performance of ridged backslabs in comparison to traditional non-ridged backslabs, specifically focusing on their load-bearing capacity and cost-effectiveness when used in lower limb fractures.

Methods

This experimental study, conducted between January 2023 and June 2023, compares three groups of backslabs with varying layers (eight, ten, and twelve) that were fabricated, each consisting of four ridged and four non-ridged specimens. These backslabs, constructed from six-inch plaster of Paris rolls, were 190 cm in length. A three-point bending test was conducted on both groups using a Hounsfield H100KS Universal Testing Machine (Tinius Olsen Ltd., Redhill, UK), with a crosshead speed of 5 mm/min and a span distance of 190 mm between supports.

Results

Significant differences in mean maximum force endured were observed between the ten-layered and twelve-layered flat and ridged backslabs (p-values: 0.003 and 0.004, respectively). Ten-layered ridged backslabs exhibited a 56 N higher load-bearing capacity, while twelve-layered ridged backslabs withstood 73.9 N more force than their flat counterparts, underscoring the superior strength of ridged lower limb backslabs.

Conclusion

Ridged backslabs outperformed non-ridged backslabs in terms of strength when subjected to external forces. These findings support the potential adoption of ridged backslabs as a lightweight, cost-effective, and robust alternative for immobilization in lower limb fractures.

## Introduction

Lower limb backslabs are frequently used in the initial management to treat nondisplaced fractures, stabilize ankle fractures temporarily while waiting for surgery, or manage non-displaced fractures after lower limb surgery to maintain a plantigrade posture and avoid contracture [1]. The three-point casting loading principle was first outlined by Charnley for the treatment of fractures [2]. A potentially fatal risk of compartment syndrome is linked to fractures and subsequent immobilization in a constricting cast. Compartment syndrome is brought on by increased pressures in a restricted space that obstructs the arterial blood supply to muscles [1]. As a result, applying a backslab rather than complete casts has become standard procedure to eliminate this risk.

According to fundamental orthopaedic principles, if a fracture occurs below the elbow or knee, it should be immobilized distal to the injury and, ideally, both joints should be immobilized proximally and distally. However, different methods used while applying the backslabs initially can lead to weak plasters that could break or be unsuccessful at immobilizing, which is frequently highlighted during the review [3]. Fractures may move, which can cause the sufferer needless suffering [3].

Rationale

Lower limb fractures are a prevalent form of injury, and the utilization of backslabs for immobilization is a well-accepted and customary therapeutic approach. Nevertheless, conventional backslabs are characterized by their substantial size, lack of comfort, and limited efficacy in terms of immobilization. Ridged backslabs represent a more recent iteration of backslabs, potentially presenting a range of advantages when compared to conventional backslabs. They are lighter, more comfortable, and potentially more efficacious in mitigating motion. The primary objective of this research endeavor was to conduct a comparative analysis of the efficacy of ridged and non-ridged backslabs in the context of immobilizing fractures in the lower limb.

Significance

The strength of plaster splints can be enhanced by incorporating a longitudinal ridge, as opposed to increasing the amount of material used in the splint [3-6]. Numerous researchers have conducted studies on advancements in splinting techniques with the aim of enhancing the longevity of splints and optimizing the utilization of splinting materials (4-6). As per the author's knowledge, this study is the first study comparing the mechanical properties of non-ridged versus ridged backslabs in lower limb fractures. It has the potential to significantly impact therapeutic interventions, improving patient outcomes and reducing healthcare costs. The use of ridged backslabs could become the standard of care, offering advantages such as reduced discomfort, enhanced range of motion, and improved functionality. Additionally, the cost-effectiveness of ridged backslabs, attributed to their lighter weight and simpler manufacturing process, may further contribute to their adoption over conventional backslabs.

## Materials and methods

**Figure 1:**
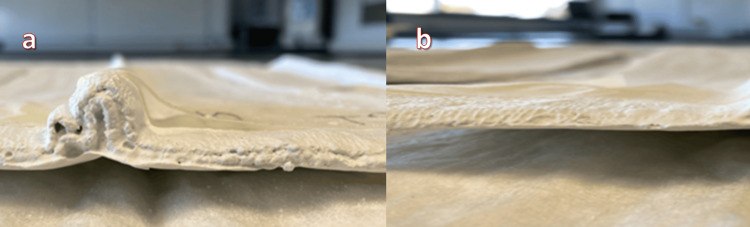
Plaster of Paris ridged backslabs (a) and non-ridged backslabs (b)

In this experimental study conducted between January 2023 and June 2023, plaster of Paris backslabs of eight, ten, and twelve layers were constructed, with each group consisting of four ridged (Figure 1a) and four non-ridged backslabs (Figure 1b).

**Figure 2:**
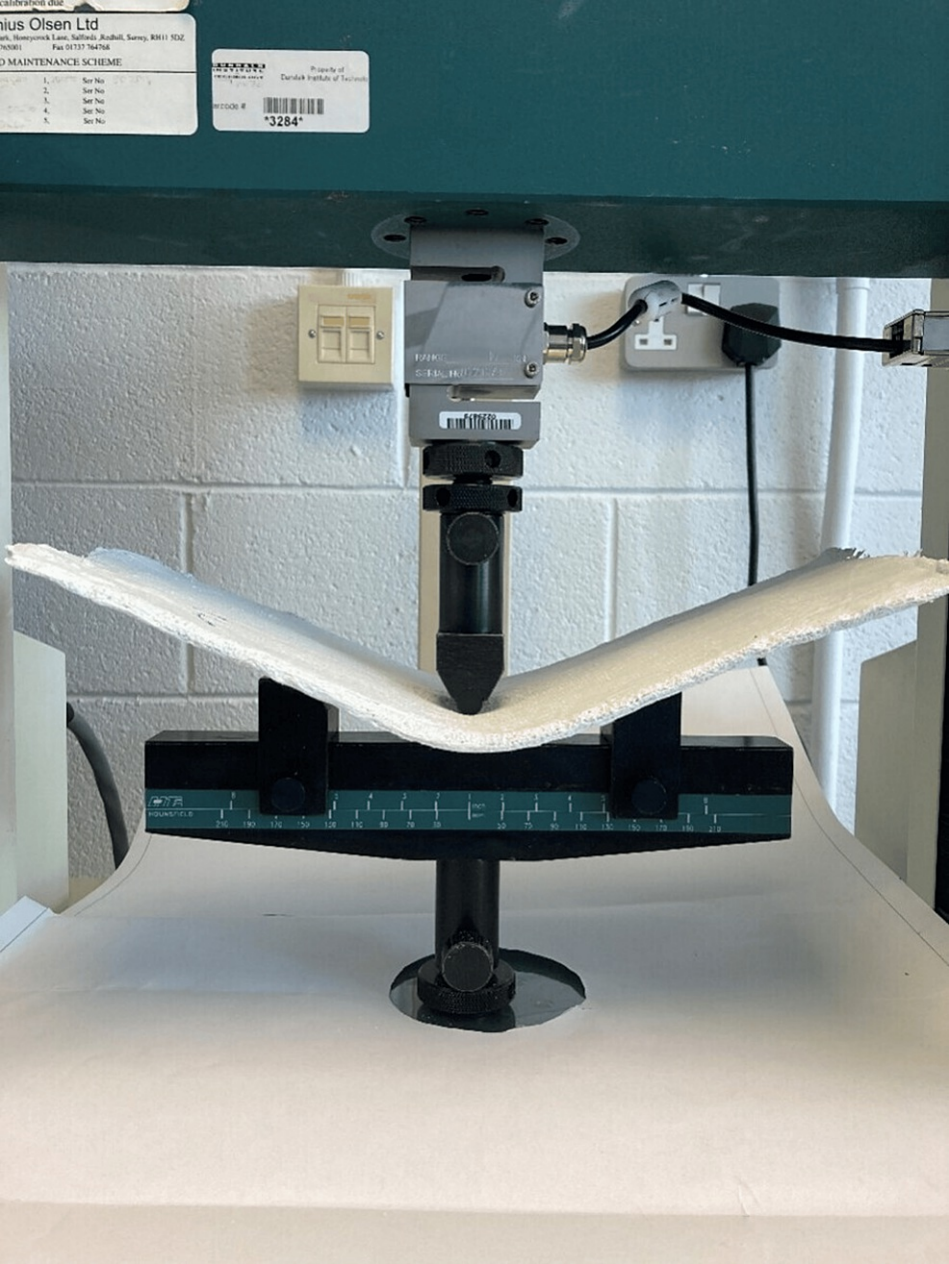
Three-point bending using a Hounsfield H100KS Universal Testing Machine Courtesy: Materials Lab, Department of Electronic and Mechanical Engineering, Institute of Technology, Dundalk, Ireland.

The backslabs were 5 mm thick with 35 mm in width. They were left to dry for 24 hours. All three specimen groups were subjected to a three-point bending using a Hounsfield H100KS Universal Testing Machine (Tinius Olsen Ltd., Redhill, UK) at a crosshead speed of 5 mm/min and a span distance between supports of 190 mm (Figure 2). The maximum load at failure was recorded using a 10 kN load cell with a resolution of 0.1 N. The results were plotted in a tabulated form and bar chart.

A comprehensive statistical analysis was conducted in IBM SPSS version 26 (IBM Corp., Armonk, USA) to investigate the maximum load-bearing capacities exhibited by both flat and ridged backslabs. The central tendency and variability of these load-bearing capacities were quantified through the computation of the mean and standard deviation, respectively. In addition, the normality of the data distribution was assessed employing the Shapiro-Wilk test, with a p-value <0.05 as significant. A p-value exceeding 0.05 was adopted as indicative of a normal distribution of the data. To explore potential disparities in load-bearing capabilities between the two distinct types of slabs, an independent sample t-test was employed. This analytical approach facilitated the assessment of whether any observed differences in load to failure were statistically significant.

## Results

After analyzing the data obtained from both the flat and ridged backslabs, it was found that there was a mean difference with respect to load to failure (Table 1). In the "Eight layered flat backslabs," the average load was 164.41 N with a standard deviation of 14.21 N, while the "Eight layered ridged backslabs" exhibited a higher average load of 182.85 N with a standard deviation of 13.37 N. Similarly, in the "Ten layered flat backslabs," the average load was 226.75 N with a standard deviation of 10.33 N, whereas the "Ten layered ridged backslabs" had an even higher average load of 283 N with a standard deviation of 21.24 N. Finally, in the "Twelve layered flat backslabs," the average load was 301 N with a standard deviation of 17.09 N, and the "Twelve layered ridged backslabs" showed the highest average load of 374.97 N with a standard deviation of 27.44 N. These data collectively demonstrate that ridged backslabs consistently exhibited a superior load-bearing capacity compared to flat backslabs across different layer configurations, suggesting the potential advantages of ridged backslabs in terms of load-bearing capabilities.

**Table 1:** Measurements of load to failure of flat and ridged backslabs N: newton, Mean: measurement of mean load failure in newton, SD: standard deviation

Specimen number	Eight layered flat backslabs (N)	Eight layered ridged backslabs (N)	Ten layered flat backslabs (N)	Ten layered ridged backslabs (N)	Twelve layered flat backslabs (N)	Twelve layered ridged backslabs (N)
1	157.6	166.4	228.75	254.8	277.2	381.6
2	148.05	184.6	214.25	299.2	312.4	403.5
3	172.6	181.4	224.75	299.6	300	377.2
4	179.4	199	239.25	278.4	314.4	337.6
Mean ± SD	164.41 ± 14.21	182.85 ± 13.37	226.75 ± 10.33	283 ± 21.24	301 ± 17.09	374.97 ± 27.44

**Figure 3:**
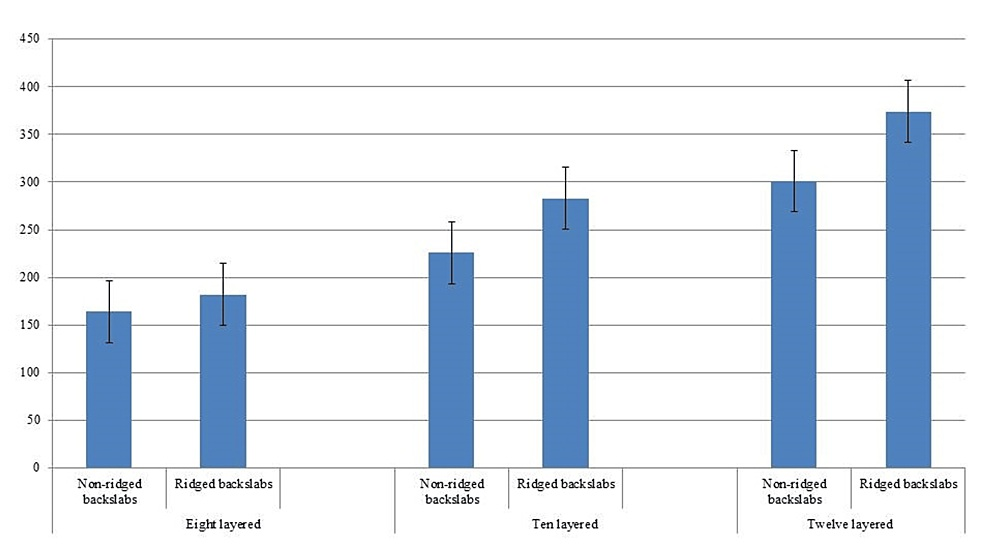
Backslab bend test results Comparison of mean measurements in Newton

Furthermore, the non-ridged backslabs could not hold on to the maximum load whereas ridged slabs especially ten- and twelve-layered showed good bend test results. These findings are visually illustrated in Figure 3, providing a graphical representation of the observed differences in load-bearing capabilities between the two types of slabs.

The final analysis as shown in Table 2 demonstrates that there was a significant difference in mean maximum force endured between ten-layered and twelve-layered non-ridged and ridged backslabs (p-value for 10-layered slabs: 0.003; p-value for 12-layered slabs: 0.004). Additionally, the ten-layered ridged backslab sustained 56 N, and the twelve-layered ridged backslab sustained 73.9 N more force than the non-ridged backslabs, which proved that the ridged lower limb backslabs are stronger.

**Table 2:** Comparison of mean differences and p-values for different layered slabs in load sustainment Mean: Mean Load Sustainment in Newton, SD: Standard Deviation, P-value <0.05 was considered significant

Type of Slabs	Mean ± SD	P-value
8 Layered	0.108 ± 18.43	0.608
10 Layered	0.003 ± 56.25	0.159
12 Layered	0.004 ± 73.97	0.54

## Discussion

Open fractures of the lower limb, a common occurrence in orthopedic emergencies, present formidable challenges in their management, characterized by a heightened susceptibility to complications and less-than-optimal clinical outcomes [7]. In recent years, the adoption of secondary nailing has emerged as a promising and effective therapeutic strategy for addressing these fractures. This approach boasts several distinct advantages, including the early restoration of weight-bearing capability and the simultaneous reestablishment of essential mechanical stability within the affected limb [8, 9].

Our investigation focused on a comparative analysis of the strength between ridged and flat backslabs, revealing the superior strength of ridged backslabs. Notably, the ten- and twelve-layered ridged backslabs exhibited statistically significant mean differences, demonstrating their capacity to endure greater applied force compared to their flat counterparts with equivalent layers. Our findings indicate that ridged backslabs consistently outperformed flat backslabs in terms of load-bearing capabilities, corroborating similar trends observed in previous studies [10, 11].

Our research findings are consistent with several prior studies that have explored the advantages of ridged backslabs in terms of load-bearing capabilities. Khan et al. [12] observed increased support from ridged backslabs during forearm immobilization in their simple breaking strain tests. Our study similarly demonstrates that ridged backslabs, particularly in the ten- and twelve-layered configurations, provide enhanced strength compared to conventional flat slabs. Stewart [13] also contributed to the understanding of ridged splints, highlighting their ability to significantly strengthen volar wrist slabs. They noted that ridges perpendicular to the major volar slab increased stability, which aligns with our findings of superior load-bearing capacity in ridged backslabs. This indicates a consistent trend in the mechanical advantages of ridged configurations.

Furthermore, the use of ridged slabs in the upper limb, as supported by previous research [4], has shown promising results. Adding anterior ridges to standard plaster of Paris (POP) volar slabs substantially increased their strength, making it easier to maintain the injured or recovering hand in the required position. This corresponds to our findings, suggesting that ridged backslabs can offer increased strength and stability, particularly in load-bearing scenarios. The study that demonstrated ridged POP volar slabs' superiority over non-ridged slabs [6] further supports our results. This consistency in findings across different studies reinforces the notion that ridged configurations provide enhanced structural strength, a factor that could be beneficial in clinical practice.

Lastly, the study [14] highlighting the impact of material, thickness, and longitudinal ridging on splint strength further underscores the potential advantages of ridged designs. Their observation that adding a longitudinal ridge to plaster splints significantly increased their strength is in line with our research, which shows that ridged backslabs, particularly in the ten- and twelve-layered forms, exhibit superior load-bearing capacity.

The current research findings corroborate and extend the existing body of evidence that supports the advantages of ridged reinforcement technology in orthopedic applications. Previous studies [15, 16] have consistently demonstrated that ridged configurations can withstand significantly higher mean loads compared to their non-ridged counterparts before deforming. Our study aligns with these findings, as we also observed that ridged backslabs exhibit superior load-bearing capabilities in the lower limb, especially in ten- and twelve-layered forms.

The utility of ridged plastering techniques in emergency nursing, as highlighted in previous research [3, 17], is indeed noteworthy. These techniques are recognized for their simplicity, adaptability, affordability, and ease of acquisition. Our findings further underscore their effectiveness by demonstrating that ridged backslabs can maintain alignment and increase patient compliance, two critical factors in fracture management. This aligns with the concept of continuity of care, as ridged backslabs can contribute to ensuring that patients receive optimal care and support for their fractures. Moreover, the ability of ridged configurations to provide analgesia is consistent with the observed advantages in our study. By offering enhanced structural stability and support, ridged backslabs may help alleviate discomfort and pain associated with fractures. This aligns with the concept of maintaining patient comfort and minimizing distress during the healing process.

The foremost limitation of the present study was that it was not performed on human subjects, but the strength of the study was that this was the first of the studies to be done to check ridged lower limb backslab strength. This is certainly important for future researchers as the lower limb has not been a center of attention for ridged backslab.

## Conclusions

The study's findings emphasize the remarkable load-bearing capacity of ridged backslabs compared to traditional non-ridged counterparts. Significantly higher strength was observed in both ten-layered and twelve-layered configurations, with ten-layered ridged backslabs exhibiting a 56 N increase and twelve-layered ridged backslabs withstanding an impressive 73.9 N more force than flat counterparts. These outcomes highlight the undeniable advantages of ridged backslabs, including enhanced immobilization effectiveness, reduced material usage, and improved cost-effectiveness. Our recommendation based on these findings is the adoption of a minimum of ten layers of ridged backslabs for conservative treatment of lower limb fractures, ensuring superior strength, patient comfort, and cost-efficiency in clinical practice.
